# Phytotherapies in motion: French Guiana as a case study for cross-cultural ethnobotanical hybridization

**DOI:** 10.1186/s13002-020-00404-1

**Published:** 2020-09-16

**Authors:** M.-A. Tareau, A. Bonnefond, M. Palisse, G. Odonne

**Affiliations:** 1 LEEISA (Laboratoire Ecologie, Evolution, Interactions des Systèmes Amazoniens), CNRS, Université de Guyane, IFREMER, 97300 Cayenne, French Guiana; 2 Cayenne, French Guiana

**Keywords:** Cultural keystone species, Indicator species, Exchange networks, Migrations

## Abstract

**Background:**

French Guiana is characterized by a very multicultural population, made up of formerly settled groups (Amerindians, Maroons, Creoles) and more recent migrants (mostly from Latin America and the Caribbean). It is the ideal place to try to understand the influence of intercultural exchanges on the composition of medicinal floras and the evolution of phytotherapies under the effect of cross-culturalism.

**Methods:**

A combination of qualitative and quantitative methods was used. Semi-directive interviews were conducted in 12 localities of French Guiana’s coast between January 2016 and June 2017, and the responses to all closed questions collected during the survey were computerized in an Excel spreadsheet to facilitate quantitative processing. Herbarium vouchers were collected and deposited at the Cayenne Herbarium to determine Linnaean names of medicinal species mentioned by the interviewees. A list of indicator species for each cultural group considered was adapted from community ecology to this ethnobiological context, according to the Dufrêne-Legendre model, via the “labdsv” package and the “indval” function, after performing a redundancy analysis (RDA).

**Results:**

A total of 205 people, belonging to 15 distinct cultural groups, were interviewed using semi-structured questionnaires. A total of 356 species (for 106 botanical families) were cited. We observed that pantropical and edible species hold a special place in these pharmacopeias. If compared to previous inventories, 31 recently introduced species can be counted. Furthermore, this study shows that the majority of the plants used are not specific to a particular group but shared by many communities. However, despite this obvious cross-culturalism of medicinal plants between the different cultural communities of French Guiana, divergent trends nevertheless appear through the importance of 29 indicator/cultural keystone species in 10 cultural groups. Finally, we have emphasized that the transmission of herbal medicine’s knowledge in French Guiana is mainly feminine and intra-cultural.

**Conclusion:**

French Guianese medicinal flora is undoubtedly related to the multiple cultures that settled this territory through the last centuries. Cultural pharmacopeias are more hybrid than sometimes expected, but cultural keystone species nevertheless arise from a common background, allowing to understand, and define, the relationships between cultural groups.

## Background

Ethnobotany of migrants is occupying an increasingly important place in modern ethnobiology, thanks to numerous recent works of ethnographic description and conceptualization of these practices [1–13], and particularly in an urban context where moving populations are often concentrated [1, 2, 5, 8–10, 14–19]. Indeed, the question of the resulting ethnobotanical hybridizations is now at the heart of interdisciplinary questioning in ethnosciences [20–25], as it is nowadays accepted that phytotherapeutical practices are dynamic cultural processes which are constantly nourished by the circulation of people and the interactions between different cultural groups.

**Fig. 1:**
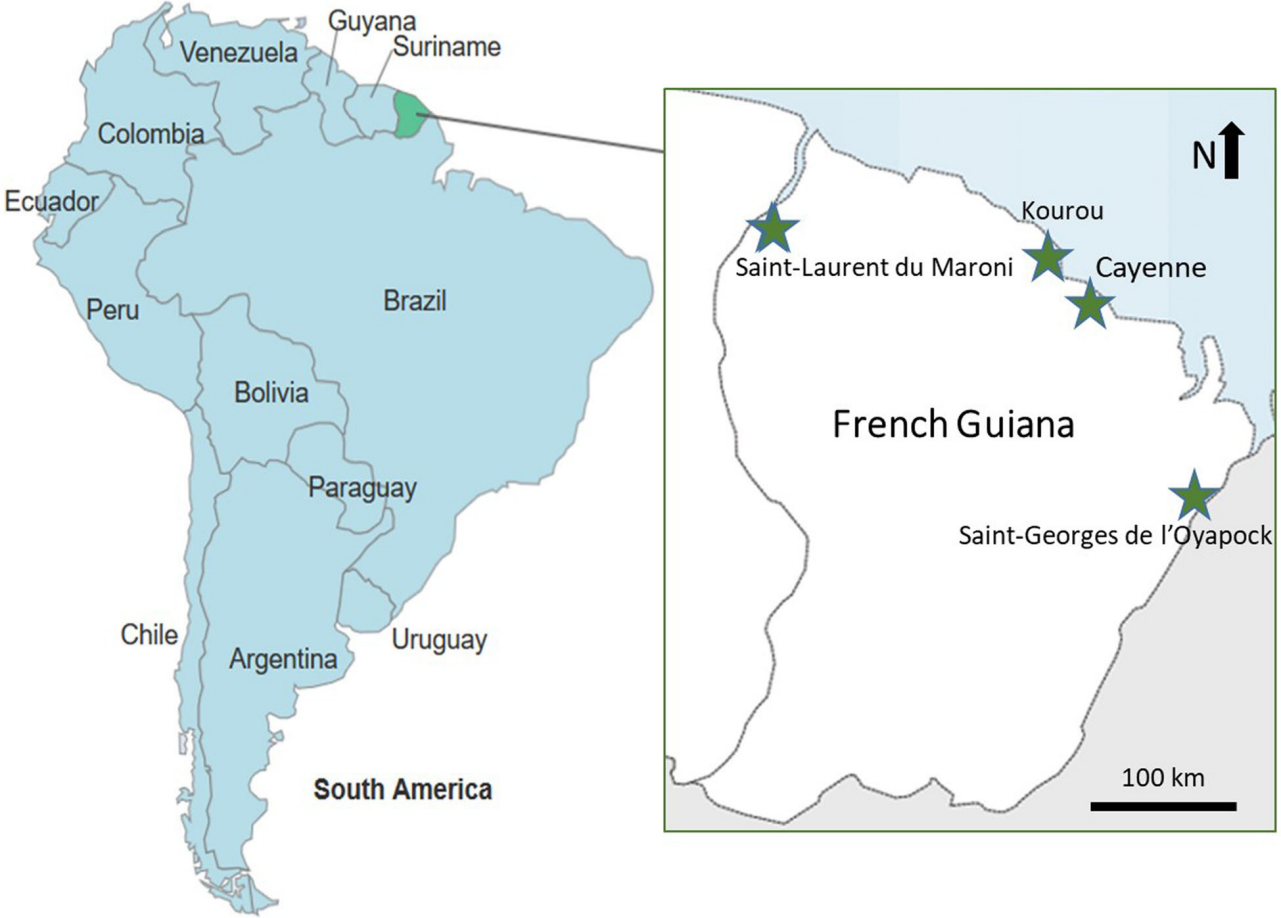
Location map of French Guiana

The highly multicultural context of French Guiana is a very interesting example to study these changes on the scale of a medium-sized area. This French territory in South America (Fig. 1), after having received many cultural groups during the colonial era, is today hosting a migrant population estimated to be more than a third of the total population (https://www.insee.fr/fr/statistiques/4177174?sommaire=4177618&geo=REG-03) counting many inhabitants originating from Haiti, Brazil, Suriname, or even the Dominican Republic and Peru, and giving rise to a fairly remarkable intercultural mix [26, 27].

If the vitality of herbal medicine practices among young French Guianese urban people has already been shown [16], along with the importance in this Amazonian space of the gathering of medicinal plants by several populations [17] and the circulation of medicinal plants at its borders, especially with Brazil [28], a general overview of the current phytotherapeutic uses in this territory had not been established yet. Thus, from a perspective seeking to question every cultural group present, we wanted to understand what could be the main dynamics in ethnopharmacopeias in this context of interculturality, migration, and urbanization, in order to better understand how they adapt to these social changes. Thus, the main questions that this study attempts to answer can be summarized as follows:

- Does multiculturalism, as found in French Guiana, favor the appearance of a multitude of pharmacopeias or, on the contrary, do the interactions between all these groups cause leveling of knowledge and practices?
- Does migratory and cross-cultural context favor greater biocultural diversity? How is the distribution of medicinal flora organized according to ethnic groups, in a situation of cultural pluralism?
- How are the transmission processes shaped in these particular contexts, in terms of groups, but also in terms of individuals (sex, age)?

## Methods

### Interviews

The data used for this article served as the ethnographic basis for the production of Tareau’s PhD thesis [29]. Interviews were realized between January 2016 and June 2017, i.e., during 18 months, over a wide strip covering the whole of the French Guiana coast (Fig. 1), comprising 12 municipalities out of the 22 municipalities of this territory. Urban people, living in the main cities of French Guiana (Cayenne, Kourou, Matoury, Rémire-Montjoly, and Saint-Laurent du Maroni), were interviewed, as were non-urban dwellers living in small countryside villages.

The first people interviewed were approached informally, on the street or at their workplace. Then, by snowball sampling [30], other respondents were gradually contacted. Semi-structured interviews were conducted, detailing the profile of each person interviewed, the medicinal species they used, the culturally specific or rather shared nature of each of them, and their access strategies and transmission patterns.

### Prior informed consent and access to biodiversity

Prior to the implementation of the French transcription of the Nagoya protocol, and in the absence of internal ethics committee at the Université de Guyane, we worked in accordance with the recommendations of the Code of Ethics of the International Society of Ethnobiology [31]. Informed consent forms were given to all the respondents in order to present and explain to them clearly the objectives of this research project and to obtain their signed agreement to participate. Each of the interviewees was informed beforehand of the confidentiality of this study, and of his/her right to withdraw its participation at any time, and of the objective of publication at the end under the form of a PhD thesis and scientific publications.

### Statistical analysis

The responses to all closed questions collected during the survey were computerized in an Excel spreadsheet to facilitate quantitative processing [32], coupled with a complementary qualitative approach.

Use reports (URs), as explained by Phillips and Gentry [33], correspond to the frequency of citations of one or more species, in general or for a particular therapeutic indication. These use reports can also be converted into percentage of the total number of uses.

Community groups were defined according to the interviewee’s stated language of first socialization, place of birth, and the cultural identity to which they say themselves that they primarily belong.

Statistical analyses were performed using R 3.3.3 [34] in order to explore if the composition of specific medicinal floras differed according to ethnicity. To do so, the global dataset was refined to make it suitable for statistical analyses. Community groups were restricted to those encompassing a minimum of 5 persons. To avoid introducing a bias according to the “level of knowledge” of each person interviewed on the topic, a post-processing sorting was carried out aiming to remove people who mentioned either much more, or fewer species compared to other (species citation per person thus range from 2 to 64 species). Then, the least cited species (< 3 URs) were excluded, considering their uses as not representative in the overall population.

The indval function in the R package “labdsv” [35] was used to set a list of indicator species, according to the Dufrêne-Legendre model, for each cultural group considered. This method is commonly used in ecology to detect indicator species in ecosystems and was adapted here to highlight indicator species in different cultural groups. In short, this method gives a maximum index when all URs of a species are found in a single cultural group and when that species is cited by all individuals belonging to that group.

A redundancy analysis (RDA) was conducted using the “*Vegan*” package [36]. Abundance data were Hellinger transformed. Significance levels were set to a nominal type-I error of 5%.

A food web analysis was performed to understand the plant exchanges among cultural groups. It has been made by considering the main intercultural fluxes of knowledge (≥ 0.5% of total URs, with *N* = 3592) and only the groups counting for more than 1% of all the transmitted URs (*N* = 3592) were conserved in the plot.

### Botany

Voucher specimens of cited plants were collected with the informants as much as possible. They were then processed and deposited at the Cayenne IRD Herbarium (CAY). Botanical determinations were performed by M. A. Tareau and G. Odonne. The taxonomical nomenclature used was the APG IV [37].

## Results

### People interviewed

A total of 205 people were interviewed using semi-structured questionnaires, belonging to 15 distinct cultural groups (Table 1).

**Table 1:** Table presenting the number of individuals questioned by community, the number of use reports (URs) provided by group, and the average per person of each group

Cultural groups	Number	URs	URs/informant
French Guianese Creoles	55	1165	21.2
Ndjuka	19	483	25.4
Haitians	19	267	14.1
French Caribbean Creoles	18	264	14.7
St Lucians	18	541	30.1
Palikur	15	175	11.6
Brazilians	15	153	10.2
Saamaka	12	161	13.4
Kali’na	10	120	12.2
French from mainland France	7	85	12.1
Guyanese	4	56	14
Aluku	4	62	15.5
Dominicans	3	38	12.6
Galibi-Marwono	3	48	16
Karipuna	3	65	21.7
**Total**	**205**	**3683**	**17**.**6**

After refining for statistical analyses, the new dataset counts 200 interviews from 12 cultural groups: French Guianese Creoles (61 persons); Aluku, Paamaka, Ndjuka and Saamaka Maroons (33 p.); Haitians (19 p.); French Caribbean Creoles (18 p.); Brazilians (15 p.); Pahikwene (12 p.); St Lucians (11 p.); Kali’na (10 p.); Europeans (6 p.); Galibi-Marwono and Karipuna (5 p.); Guyanese (5 p.); and Peruvians and Dominicans (5 p.).

Respondents have an average age of 52.5 years. Seventy-seven people were, at the time of the survey, between 18 and 40 years old (1110 URs), 92 were between 41 and 65 years old (1521 URs), and 39 were over 65 years old (1052 URs). The sampling is constituted of 58.5% of women (*N* = 120; 2355 URs) and 41.5% of men (*N* = 85; 1328 URs); 59.5% are considered urbans (*N* = 122; 2101 URs) and 40.5% non-urbans (*N* = 83; 1582 URs).

This study shows that the practice of herbal medicines is relatively active in French Guiana, since 77.5% of the people interviewed (163/205 individuals) regularly use herbal remedies (at least once every 6 months). Thus, contrary to a preconceived idea of a certain disintegration of traditional medicines linked to urbanization and a modernization of lifestyles, phytotherapeutic practices and knowledge are still alive on the French Guianese coast, and this even in the city (17.2 citations on average among city dwellers, 2101/3683 URs, 122 individuals—against 18.2 among rural dwellers, 1582/3683 URs, 87 individuals counted). Moreover, this persistent use of medicinal plants is particularly marked within certain socio-cultural groups such as St Lucians, Maroons and French Caribbean Creoles, and French Guianese Creoles, who all count more than 14 uses per informant in average (Table 1).

### Botany: what species are used?

A total of 356 species (belonging to 106 distinct families) were cited during this survey, for their medicinal, magic, or cosmetic properties, of which 212 have been collected for herbarium vouchers. Among the species for which any specimen was collected, most are very common (such as *Musa* x *paradisiaca*) and/or too difficult to process (such as *Cocos nucifera*) or do not exist in a living form in French Guiana (such as *Ferula assa-foetida*). Beyond the relatively high number of medicinal plants and the large variety of botanical families revealed by this inventory, it appears that some of them are particularly mobilized; 12 families are mainly represented, each comprising more than 100 URs. These are the Lamiaceae (218/3683 URs, for 18 species), Arecaceae (168 URs, 9 species), Fabaceae (167 URs, 22 species), Verbenaceae (164 URs, 7 species), Euphorbiaceae (153 URs, 11 species), Poaceae (147 URs, 10 species), Malvaceae (143 URs, 12 species), Asteraceae (140 URs, 27 species), Rutaceae (135 URs, 7 species), Zingiberaceae (127 URs, 6 species), and Piperaceae (107 URs, 7 species).

Among all the mentioned plants, 155 species, belonging to 62 different botanical families, are cited at least 5 times (Table 2) among which 57 species were particularly cited with more than 20 URs each. Another significant result is that, out of the 20 main species used in French Guiana, 15 can be considered as pantropical: *Allium sativum*, *Aloe vera*, *Annona muricata*, *Citrus aurantiifolia*, *Cocos nucifera*, *Cymbopogon citratus*, *Eryngium foetidum*, *Gossypium barbadense*, *Kalanchoe pinnata*, *Lippia alba*, *Ocimum basilicum*, *Momordica charantia*, *Peperomia pellucida*, *Ricinus communis*, and *Zingiber officinale*.

**Table 2:** List of the 151 main medicinal species (≥ 5 usage citations) mentioned on the coastal area of French Guiana, ranked in descending order of citations in the surveys

***Species*** (herbarium no.)	Family	Citations	Transversality (number of cultural groups concerned)	Origin	Status	Habitat
*Cymbopogon citratus* (DC.) Stapf (MAT 165)	Poaceae	113	16	AS	C	G
*Cocos nucifera* L.	Arecaceae	92	14	AS	W/C	G/R
*Citrus aurantiifolia* (Christm.) Swingle (MAT 07)	Rutaceae	85	15	AS	C	G
*Gossypium barbadense* L. (MAT 170)	Malvaceae	77	11	AM	C	G
*Quassia amara* L. (MAT 452)	Simaroubaceae	72	12	AM	W/C	G/F
*Aloe vera* (L.) Burm.f*.* (MAT 93)	Xanthorrhoeaceae	67	15	AM	C	G
*Lippia alba* (Mill.) N.E.Br. ex Britton & P.Wilson	Verbenaceae	67	11	AM	C	G
*Ricinus communis* L.	Euphorbiaceae	66	11	AF	C	G
*Siparuna guianensis* Aubl. (MAT 293)	Siparunaceae	66	12	AM	W	G/F
*Carapa guianensis* Aubl.	Meliaceae	65	12	AM	W	F/I
*Momordica charantia* L. (MAT 299)	Cucurbitaceae	60	10	AF	P/C	R/G
*Astrocaryum vulgare* Mart.	Arecaceae	58	10	AM	W	R
*Eryngium foetidum* L. (MAT 497)	Apiaceae	55	10	AS	C	G
*Annona muricata* L. (MAT 180)	Annonaceae	52	12	AM	C	G
*Allium sativum* L.	Amaryllidaceae	52	12	EU	C	I
*Peperomia pellucida* (L.) Kunth (MAT 109)	Piperaceae	49	10	AM	P	G
*Tinospora crispa* (L.) Hook. f. & Thomson (MAT 265)	Menispermaceae	48	8	AS	C	G
*Kalanchoe pinnata* (Lam.) Pers. (MAT 135)	Crassulaceae	45	13	AF	C	G
*Zingiber officinale* Roscoe	Zingiberaceae	45	11	AS	C	G
*Ocimum basilicum* L. *Ocimum campechianum* Mill. (MAT 150) *Ocimum minimum* L.	Lamiaceae	43	8	AS/AM	C/P	G/R
*Phyllanthus amarus* Schumach. & Thonn. (MAT 303)	Phyllanthaceae	43	10	AF	P	R/G
*Chenopodium ambrosioides* L. (MAT 448)	Amaranthaceae	42	7	AM	C	G
*Lantana camara* L. (MAT 298)	Verbenaceae	41	10	AM	C	G
*Citrus × aurantium* L. (MAT 498)	Rutaceae	40	9	AS	C	G
*Cinnamomum verum* J. Presl	Lauraceae	39	9	AS	C	G
*Petiveria alliacea* L. (MAT 173)	Phytolaccaceae	39	7	AM	C	G
*Piper marginatum* Jacq. (MAT 263)	Piperaceae	36	9	AM	W	R
*Senna alata* (L.) Roxb. (MAT 325) *Senna reticulata* (Willd.) H.S.Irwin & Barneby	Fabaceae	36	11	AM	W/C	R
*Annona squamosa* L. (MAT 181)	Annonaceae	35	5	AM	C	G
*Curcuma longa* L. (MAT 221)	Zingiberaceae	35	10	AS	C	G
*Costus spiralis* (Jacq.) Roscoe (MAT 205)	Costaceae	35	8	AM	W/C	R/G
*Aristolochia trilobata* L. (MAT 128)	Aristolochiaceae	34	4	AM	C	G
*Alpinia zerumbet* (Pers.) B.L. Burtt & R.M. Sm. (MAT 163)	Zingiberaceae	34	6	AS	C	G
*Ayapana triplinervis* (Vahl) R.M. King & H. Rob. (MAT 234)	Asteraceae	34	8	AM	C	G
*Anacardium occidentale* L. (MAT 194)	Anacardiaceae	33	14	AM	W/C	G/R
*Manihot esculenta* Crantz	Euphorbiaceae	33	6	AM	C	G
*Leonotis nepetifolia* (L.) R.Br.	Lamiaceae	29	9	AM	C	G
*Psidium guajava* L. (MAT 499)	Myrtaceae	29	12	AM	C	G
*Tetradenia riparia* (Hochst.) Codd (MAT 199)	Lamiaceae	28	8	AS	C	G
*Stachytarpheta jamaicensis* (L.) Vahl (MAT 174) *Stachytarpheta cayennensis* (Rich.) Vahl (MAT 271)	Verbenaceae	28	7	AM	W/C	G/R
*Scoparia dulcis* L. (MAT 295)	Plantaginaceae	27	9	AM	P	R/R
*Carica papaya* L.	Caricaceae	26	10	AM	C	G
*Hibiscus rosa-sinensis* L. (MAT 501)	Malvaceae	26	9	AS	C	G
*Senna alexandrina* Mill.	Fabaceae	26	6	AF	C	I
*Cecropia* spp.	Urticaceae	25	8	AM	W	R
*Eleutherine bulbosa* (Mill.) Urb. (MAT 223)	Iridaceae	25	6	AM	C	G
*Morinda citrifolia* L. (MAT 124)	Rubiaceae	24	8	AS	C	G
*Mangifera indica* L. (MAT 510)	Anacardiaceae	24	10	AM	C	G
*Solanum leucocarpon* Dunal (MAT 331)	Solanaceae	24	6	AM	C	G
*Justicia pectoralis* Jacq. (MAT 500)	Acanthaceae	22	5	AM	C	G
*Musa* x *paradisiaca*.	Musaceae	22	7	AS	C	G
*Portulaca oleracea* L. (MAT 107)	Portulacaceae	22	7	CO	C	G
*Plectranthus amboinicus* (Lour.) Spreng. (MAT 162)	Lamiaceae	21	8	AS	C	G
*Mansoa alliacea* (Lam.) A.H.Gentry (MAT 285)	Bignoniaceae	19	8	AM	W/C	G/F
*Senna occidentalis* (L.) Link (MAT 104)	Fabaceae	19	5	AM	C	G
*Chromolaena odorata* (L.) R.M. King & H.Rob. (MAT 228)	Asteraceae	18	8	AM	W	R
*Capraria biflora* L. (MAT 492)	Scrophulariaceae	17	5	AM	C	G
*Geissospermum* spp.	Apocynaceae	17	6	AM	W	F
*Crescentia cujete* L. (MAT 502)	Bignoniaceae	16	7	AM	C	G
*Copaifera* spp*.*	Fabaceae	16	6	AM	W	F/I
*Jatropha curcas* L. (MAT 192)	Euphorbiaceae	16	6	AM	C	G
*Jatropha gossypiifolia* L. (MAT 164)	Euphorbiaceae	15	7	AM	C	G
*Myristica fragrans* Houtt.	Myristicaceae	15	6	AS	C	G/I
*Orthosiphon aristatus* (Blume) Miq. (MAT 509)	Lamiaceae	15	5	AS	C	G
*Ocotea guianensis* Aubl. (MAT 453)	Lauraceae	15	5	AM	W	R
*Priva lappulacea* (L.) Pers. (MAT 503)	Verbenaceae	15	6	AM	W	R
*Pimenta racemosa* (Mill.) J.W.Moore (MAT 175)	Myrtaceae	15	4	AM	C	G/I
*Sambucus simpsonii* Rehder (MAT 225)	Adoxaceae	15	3	AM	C	G
*Opuntia cochenillifera* (L.) Mill. (MAT 491)	Cactaceae	14	6	AM	C	G
*Fraxinus ornus* L.	Oleaceae	13	2	EU	W	I
*Plectranthus neochilus* Schltr. (MAT 157)	Lamiaceae	13	7	AS	C	G
*Piper peltatum* L. (MAT 169)	Piperaceae	13	6	AM	W	R
*Allium cepa* L.	Amaryllidaceae	12	6	AS	C	G/I
*Dalbergia monetaria* L.f. (MAT 281)	Fabaceae	12	7	AM	W	R
*Hyptis atrorubens* Poit. (MAT 138)	Lamiaceae	12	4	AM	W	R
*Lippia micromera* Schauer (MAT 490)	Verbenaceae	12	9	AM	C	G
*Mimosa pudica* L. (MAT 266)	Fabaceae	12	5	AM	W	R
*Macfadyena unguis-cati* (L.) A.H. Gentry (MAT 233)	Bignoniaceae	12	4	AM	W	F
*Mentha* spp*.*	Lamiaceae	12	9	AS	C	G
*Nicotiana tabacum* L.	Solanaceae	12	8	AM	C	G
*Persea americana* Mill.	Lauraceae	12	4	AM	C	G
*Arrabidaea chica* (Humb. & Bonpl.) Verl. (MAT 166)	Bignoniaceae	11	5	AM	W/C	G/F
*Euterpe oleracea* Mart.	Arecaceae	11	6	AM	W	F
*Plectranthus barbatus* var. *grandis* (L.H.Cramer) Lukhoba & A.J.Paton	Lamiaceae	11	6	AS	C	G
*Pfaffia glomerata* (Spreng.) Pedersen (MAT 493)	Amaranthaceae	11	3	AM	C	G
*Sida glomerata* Cav. (MAT 155)	Malvaceae	11	3	AM	W	R
*Spondias mombin* L.	Anacardiaceae	11	6	AM	W/D	F/G
*Saccharum officinarum* L.	Poaceae	11	6	AS	C	G
*Averrhoa bilimbi* L.	Oxalidaceae	10	5	AS	C	G
*Cinnamomum camphora* (L.) J.Presl	Lauraceae	10	4	AS	C	I
*Cassia fistula* L. (MAT 489)	Fabaceae	10	3	AS	C	G
*Cannabis sativa* L. (MAT 125)	Cannabaceae	10	5	AS	C	G/I
*Euphorbia thymifolia* L. (MAT 243)	Euphorbiaceae	10	5	AM	W	R
*Marsypianthes chamaedrys* (Vahl) Kuntze (MAT 140)	Lamiaceae	10	3	AM	W	R
*Pogostemon heyneanus* Benth. (MAT 152)	Lamiaceae	10	3	AS	C	G
*Syzygium aromaticum* (L.) Merr. & L.M. Perry	Myrtaceae	10	5	AS	C	G
*Mikania congesta* DC. (MAT 186)	Asteraceae	9	5	AM	W	R
*Spondias dulcis* Parkinson	Anacardiaceae	9	4	AM	C	G
*Tabebuia serratifolia* (Vahl) G. Nicholson (MAT 317)	Bignoniaceae	9	6	AM	W	F
*Abelmoschus esculentus* (L.) Moench	Malvaceae	8	3	AF	C	G
*Bixa orellana* L.	Bixaceae	8	4	AM	C	G
*Vernonia condensata* Baker (MAT 188)	Asteraceae	8	5	AM	W	F
*Capsicum annuum* L.	Solanaceae	8	7	AM	C	G
*Cordyline fruticosa* (L.) A.Chev. (MAT 179)	Asparagaceae	8	4	AS	C	G
*Eugenia uniflora* L. (MAT 488)	Myrtaceae	8	2	AM	C	G
*Hymenaea courbaril* L.	Fabaceae	8	4	AM	W	F
*Protium heptaphyllum* (Aubl.) Marchand (MAT 229)	Burseraceae	8	4	AM	W	F
*Pilea microphylla* L. (MAT 494)	Urticaceae	8	2	AM	W	G
*Terminalia catappa* L. (MAT 504)	Combretaceae	8	5	AS	W/C	G/R
*Artocarpus altilis* (Parkinson ex F.A.Zorn) Fosberg	Moraceae	7	4	AS	C	G
*Alternanthera brasiliana* (L.) Kuntze (MAT 289)	Amaranthaceae	7	3	AM	C	G
*Elephantopus mollis* Kunth (MAT 132)	Asteraceae	7	4	AM	W	R
*Justicia secunda* Vahl (MAT 487)	Acanthaceae	7	3	AM	C	G
*Laportea aestuans* (L.) Chew (MAT 154)	Urticaceae	7	3	AM	W	R/G
*Neurolaena lobata* (L.) Cass*.*	Asteraceae	7	3	AM	C	I
*Petroselinum crispum* (Mill.) Fuss	Apiaceae	7	4	EU	C	G
*Passiflora edulis* Sims	Passifloraceae	7	6	AM	C	G
*Cyperus odoratus* L. (MAT 248)	Cyperaceae	7	3	AM	C	G/R
*Sphagneticola trilobata* (L.) Pruski (MAT 226)	Asteraceae	7	3	AM	C	G/R
*Begonia glabra* Aubl. (MAT 297)	Begoniaceae	6	3	AM	W/D	F/G
*Campomanesia aromatica* (Aubl.) Griseb.	Myrtaceae	6	3	AM	W	R
*Clidemia hirta* (L.) D. Don (MAT 159)	Melastomataceae	6	4	AM	W	R
*Caesalpinia pulcherrima* (L.) Sw. (MAT 508)	Fabaceae	6	2	AF	C	G
*Catharanthus roseus* (L.) G. Don	Apocynaceae	6	3	AF	C	G
*Coutoubea spicata* Aubl. (MAT 127)	Gentianaceae	6	3	AM	W	R
*Cucurbita maxima* Duchesne	Cucurbitaceae	6	2	AM	C	G
*Dipteryx odorata* (Aubl.) Willd.	Fabaceae	6	2	AM	W	F
*Euphorbia hirta* L. (MAT 495)	Euphorbiaceae	6	3	AS	W	F/I
*Ferula assa-foetida* L.	Apiaceae	6	3	AF	C	I
*Inga* spp.	Fabaceae	6	2	AM	C	G
*Moringa oleifera* Lam. (MAT 507)	Moringaceae	6	5	AS	C	G
*Polyscias scutellaria* (Burm.f.) Fosberg (MAT 505)	Araliaceae	6	3	AM	C	G
*Renealmia* sp.	Zingiberaceae	6	3	AM	W/D	F/G
*Syzygium malaccense* (L.) Merr. & L.M. Perry (MAT 137)	Myrtaceae	6	3	AS	C	G
*Theobroma cacao* L.	Malvaceae	6	3	AM	C	G
*Zea mays* L.	Poaceae	6	3	AM	C	G
*Ageratum conyzoides* L. (MAT 231)	Asteraceae	5	2	AM	W	R
*Aframomum melegueta* K. Schum. (MAT 334)	Zingiberaceae	5	2	AF	C	I
*Artemisia* spp.	Asteraceae	5	3	EU	C	G
*Bidens cynapiifolia* Kunth (MAT 275)	Asteraceae	5	3	AM	W	R
*Cajanus cajan* (L.) Millsp. (MAT 486)	Fabaceae	5	2	AF	C	G
*Cordia curassavica* (Jacq.) Roem. & Schult. (MAT 300)	Boraginaceae	5	3	AM	W	R
*Commelina erecta* L. (MAT 310)	Commelinaceae	5	4	AM	C	G
*Coffea* spp.	Rubiaceae	5	3	AF	C	I
*Tanaecium bilabiatum* (Sprague) L.G. Lohmann	Bignoniacée	5	2	AM	W	F
*Malpighia emarginata* DC (MAT 496)*.*	Malpighiaceae	5	4	AM	C	G
*Plantago major* L. (MAT 506)	Plantaginaceae	5	2	EU	C	G
*Picrolemma sprucei* Hook. f.	Simaroubaceae	5	4	AM	C	F
*Rolandra fruticosa* (L.) Kuntze (MAT 203)	Asteraceae	5	3	AM	W	R
*Struchium sparganophorum* (L.) Kuntze (MAT 101)	Asteraceae	5	2	AS	C	G
*Averrhoa carambola* L.	Oxalidaceae	5	1	AS	C	G

“Origin”: *AS* Asia, *AM* America, *AF* Africa, *EU* Europe, *CO* cosmopolitan. “Status”: *C* cultivated, *W* wild, *P* protected, *D* domesticated. “Habitat”: *G* garden, *F* forest, *R* ruderal, *I* imported

In addition, edible species hold a special place in migrants’ pharmacopeias (in our sample of respondents, these are Brazilian, Dominican, Haitian, Guyanese, Peruvian, and Surinamese informants) since they represent more than a third of the species used by migrants (34.0%; 177/521 URs) and one fifth for the whole population (20.4%; 751/3683).

Finally, it is to be noted that, if compared to previous inventories [38, 39], 31 “new” species can be counted including 22 living plants: *Alpinia zerumbet*, 34 URs; *Artemisia absinthium*, 4 URs; *Blighia sapida*, 1 UR; *Cannabis sativa*, 10 URs; *Guazuma ulmifolia*, 2 URs; *Lippia micromera*, 12 URs; *Melaleuca quinquenervia*, 3 URs; *Morinda citrifolia*, 24 URs; *Moringa oleifera*, 6 URs; *Petroselinum crispum*, 7 URs; *Pimenta racemosa*, 15 URs; *Plectranthus grandis*, 11 URs; *Plectranthus neochilus*, 13 URs; *Polyscias scutellaria*, 6 URs; *Porophyllum ruderale*, 2 URs; *Punica granatum*, 2 URs; *Scutellaria purpurascens*, 2 URs; *Tanaecium bilabiatum*, 5 URs; *Tetradenia riparia*, 28 URs; *Theobroma grandiflorum*, 1 UR; *Vernonia condensata*, 8 URs; and *Syzygium malaccense*, 6 URs.

### Cross-culturalism: a widely shared medicinal flora

**Fig. 2:**
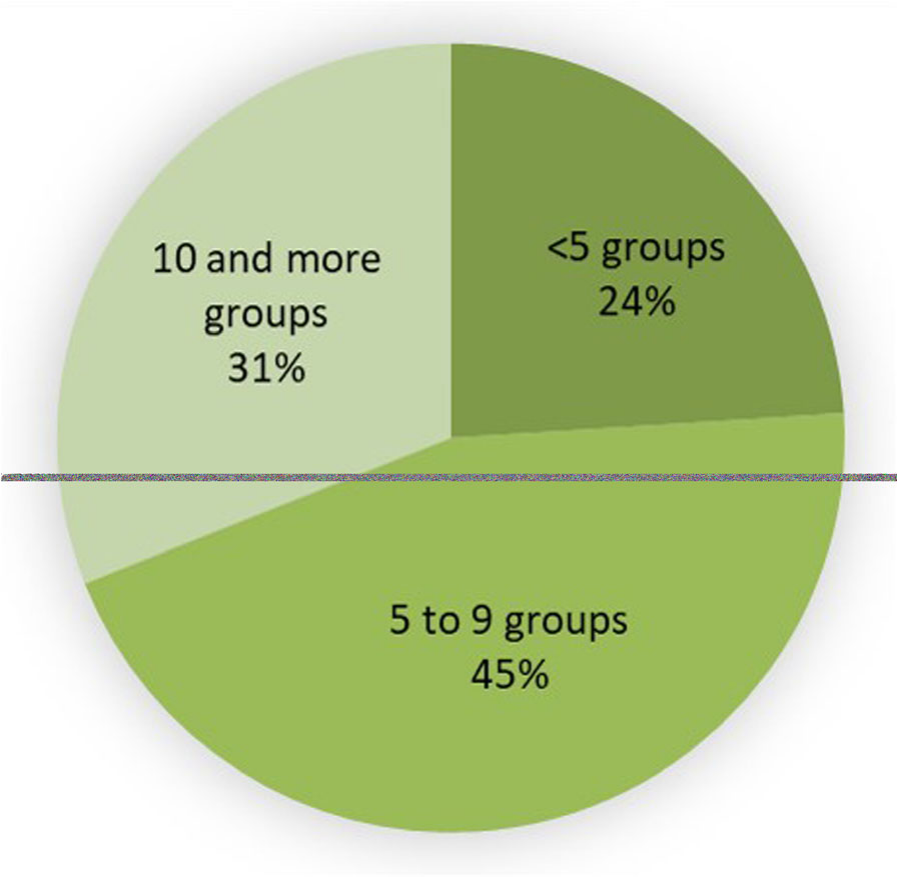
Distribution of species (in percentage of species) according to the number of cultural groups using them

This study highlights the diversity of medicinal plants in use on the French Guianese coast and shows that the majority of the plants used are not specific to a particular group but shared by many communities. More than ¾ of the species are cited by at least 5 of the cultural groups questioned during the survey, and 31.3% of them are used in more than 10 among the 15 groups questioned (Fig. 2). Only one species appears to be exclusive to a single community (*Averrhoa carambola*, cited only by French Guianese Creoles), but due to the low number of URs (only 4), it does not appear as an indicator. Community groups and individuals do not automatically consider each plant for the same use but it means at least that therapeutic properties are recognized for this plant within different communities. This does not mean that these groups use them for the same uses, but that they know these plants and their therapeutic properties.

Some cross-cultural species (*Aloe vera*, *Citrus aurantiifolia*, *Cymbopogon citratus*, *Ricinus communis*, *Zingiber officinale*…) are considered as true panaceas whose miscellaneous purposes uses are widely disseminated in the whole population, making them constitutive of a widely shared medicinal flora.

### Divergences and specificities: “Show me which plants you use, I will tell you who you are”

However, despite these shared uses of medicinal plants among the different cultural communities of French Guiana, divergent trends nevertheless appear. The importance of cultural keystone species [40] to defining cultural identities is acknowledged in ethnobotany, but detecting them in a multicultural context might be difficult. To do so, a statistical analysis borrowed from community ecology was performed as described in the “Methods” section.

This analysis led to the identification of 29 indicator/cultural keystone species in 10 cultural groups from the 12 considered (*cf.* “Methods” section), as no species were highlighted for two of them: kra and plk (Table 3). In the table, the higher the value of the *Indval* index is and the lower the *p* value is, the more closely related the indicator species is with the cultural group associated.

**Table 3:** List of the 29 indicator species associated with 10 cultural groups from French Guiana and classified according to the decreasing Indval index

***Species*** Family	Indval	URs	***P*** value	Associated groups
*Allium cepa* L. Amaryllidaceae	0.430	11	0.002**	Peruvians and Dominicans
*Vernonia condensata* Baker Asteraceae	0.393	8	0.003**	Galibi-Marwono et Karipuna
*Carapa guianensis* Aubl. Meliaceae	0.354	57	0.001***	Galibi-Marwono et Karipuna
*Persea americana* Mill. Lauraceae	0.349	11	0.001***	Guyanese
*Momordica charantia* L. Cucurbitaceae	0.337	51	0.001***	Haitians
*Lippia micromera* Schauer verbenaceae	0.311	11	0.004**	Europeans
*Picrolemma sprucei* Hook. f. Simaroubaceae	0.310	4	0.011*	Galibi-Marwono et Karipuna
*Wedelia trilobata* (L.) Hitchc. Asteraceae	0.306	5	0.011*	Guyanese
*Petiveria alliacea* L. Phytolaccaceae	0.302	31	0.003**	Galibi-Marwono et Karipuna
*Astrocaryum vulgare* Mart. Areceaceae	0.292	42	0.002**	Galibi-Marwono et Karipuna
*Abelmoschus esculentus* (L.) Moench Malvaceae	0.291	8	0.006**	Guyanese
*Mansoa alliacea* (Lam.) A.H. Gentry Bignoniaceae	0.290	14	0.005**	Galibi-Marwono et Karipuna
*Cecropia* spp. Urticaceae	0.255	20	0.011*	Guyanese
*Jatropha gossypifolia* L. Euphorbiaceae	0.237	12	0.011*	Galibi-Marwono et Karipuna
*Catharanthus roseus* (L.) G. Don Apocynaceae	0.212	6	0.038*	Kali’na
*Fraxinus ornus* L. Oleaceae	0.197	12	0.034*	French Guianese Créoles
*Passiflora edulis* Sims Passifloraceae	0.196	4	0.009**	Peruvians and Dominicans
*Dalbergia monetaria* L. f. Fabaceae	0.188	11	0.039*	Galibi-Marwono et Karipuna
*Piper peltatum* L. Piperaceae	0.183	11	0.049*	St Lucians
*Annona squamosa* L. *Bromeliaceae*	0.182	29	0.047*	St Lucians
*Chenopodium ambrosioides* L. Amaranthaceae	0.179	38	0.024*	Brazilians
*Phyllanthus amarus* Schumach. & Thonn. Phyllanthaceae	0.179	40	0.029*	St Lucians
*Tanaecium bilabiatum* (Sprague) L.G. Lohmann Bignoniaceae	0.167	5	0.030*	Aluku, Paamaka, Ndjuka, Saamaka Maroons
*Struchium sparganophorum* (L.) Kuntze Asteraceae	0.167	5	0.031*	Aluku, Paamaka, Ndjuka, Saamaka Maroons
*Plectranthus amboinicus* (Lour.) Spreng. Lamiaceae	0.159	18	0.042*	Peruvians and Dominicans
*Plantago major* L. Plantaginaceae	0.143	5	0.033*	St Lucians
*Caesalpinia pulcherrima* (L.) Sw. Fabaceae	0.143	5	0.039*	St Lucians

*significant (*p* value comprised between 0.05 and 0.01)

**highly significant (*p* value comprised between 0.01 and 0.001)

***very highly significant (*p* value under 0.001 included)

A redundancy analysis (RDA) model was built with cultural groups as predictive factors and (a) the whole species dataset suitable for analysis and (b) the set of 205 observations of indicator species. In both cases, the difference of the RDA model built with cultural as predictive with a null model (H0: the species distribution in pharmacopeias of the different persons interviewed is due to randomness) is significant. The percentage of the variation observed in species distribution explained by socio-cultural origin varies from 7.4% (a) to 16.6% (b) when considering only indicator species. While this rate reveals that cultural groups influence pharmacopeias species composition, the fact remains that other variables (age, sex, place of life, etc.) might also play important roles.

**Fig. 3:**
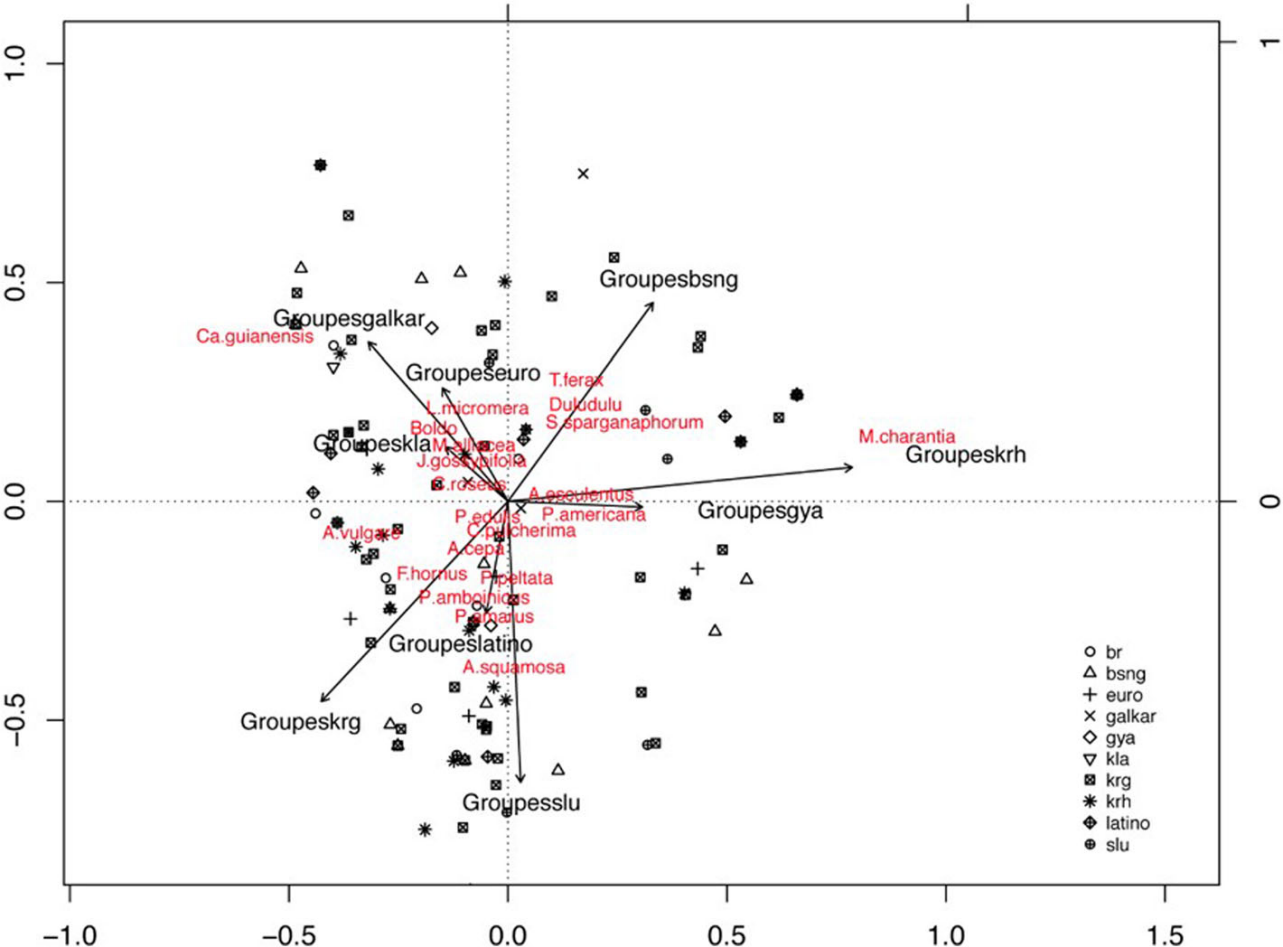
Graphical representation of the RDA model allowing to visualize the association between 29 indicator species obtained and 10 cultural groups from French Guiana. “br” = Brazilians; “bsng” = Maroons; “euro” = Europeans; “galkar” = Galibi and Karipuna; “gya” = Guyanese; “kla” = Kali’na; “krg” = French Guianese Creoles; “krh” = Haitians; “latino” = Dominicans and Peruvians; “slu = St Lucians

The triplot below (Fig. 3) is a representation of the constrained RDA model allowing to visualize the relative position of indicator species among the different groups in a virtual space.

We observe first of all that the group including the Amerindians Karipuna and Galibi-Marwono (galkar) is the one that is affected by the largest number of indicator species (8 species out of 29). In fact, these are species widely used in Brazil [41] and by the inhabitants of eastern French Guiana, border with Brazil (in particular, *Carapa guianensis*, *Dalbergia monetaria*, *Mansoa alliacea*, *Picrolemma sprucei*, *Vernonia condensata*), where the majority of the individuals of the two aforementioned groups live. However, statistical analysis may have given them disproportionate weight given the numerical differences between the groups interviewed.

All the results are consistent with the qualitative observations made in the field in terms of culturally marked practices. Some keystone species, much valued by certain cultural communities, do not necessarily appear as indicators and some are more transverse than it seems from the discourses. This is the case, for example, of *Quassia amara* whose attempts at cultural appropriation by certain communities which does not emerge as an indicator species of any particular cultural group from French Guiana, meaning that nowadays it is used by several groups, precisely 8 out of the 12 groups considered. Moreover, some species indicative of local groups are coming from distant regions, like *Fraxinus ornus* of which the exudate is used by the French Guianese Créoles as an ingredient in purgative cures, or *Catharanthus roseus*, native to the Indian Ocean, which stands out as being the only indicator species for the group of Kali'na Amerindians. Finally, Latin American migrants preferentially use cultivated pantropical food species (*Allium cepa*, *Passiflora edulis*, *Plectranthus amboinicus*), while local (and wild) species are more widely used by long-established cultural groups (Amerindians and Maroons), who are often farmers and therefore have a daily connection with the surrounding flora.

### Access strategies and transmission

The analysis of the responses to the semi-structured interviews carried out made it possible to measure the proportion of the different modes of access to medicinal plants on the French Guiana coast. We observe that 39.3% of the plants used are cultivated by the users themselves (1448/3683) and 31.6% come from picking, either in forested or urban areas (1163/3683 URs), and 20.4% are purchased commercially (752/3683). The networks of exchanges between relatives (family and acquaintances: friends, neighbors, and colleagues) total 6.0% of citations (222 citations, of which 140 plants were given by a relative and 82 by an extra-familial acquaintance). In addition, another equally significant result is that 98 URs (2.7% of total citations) come directly from outside French Guiana. These plants are generally brought by individuals (38 from Brazil, 26 from Suriname, 12 from Martinique, 9 from Haiti, 6 from St Lucia, 4 from Dominican Republic, 3 from Guadeloupe) who also bring with them processed products, like castor oil from the West Indies or *Carapa guianensis* oil from Brazil.

**Fig. 4:**
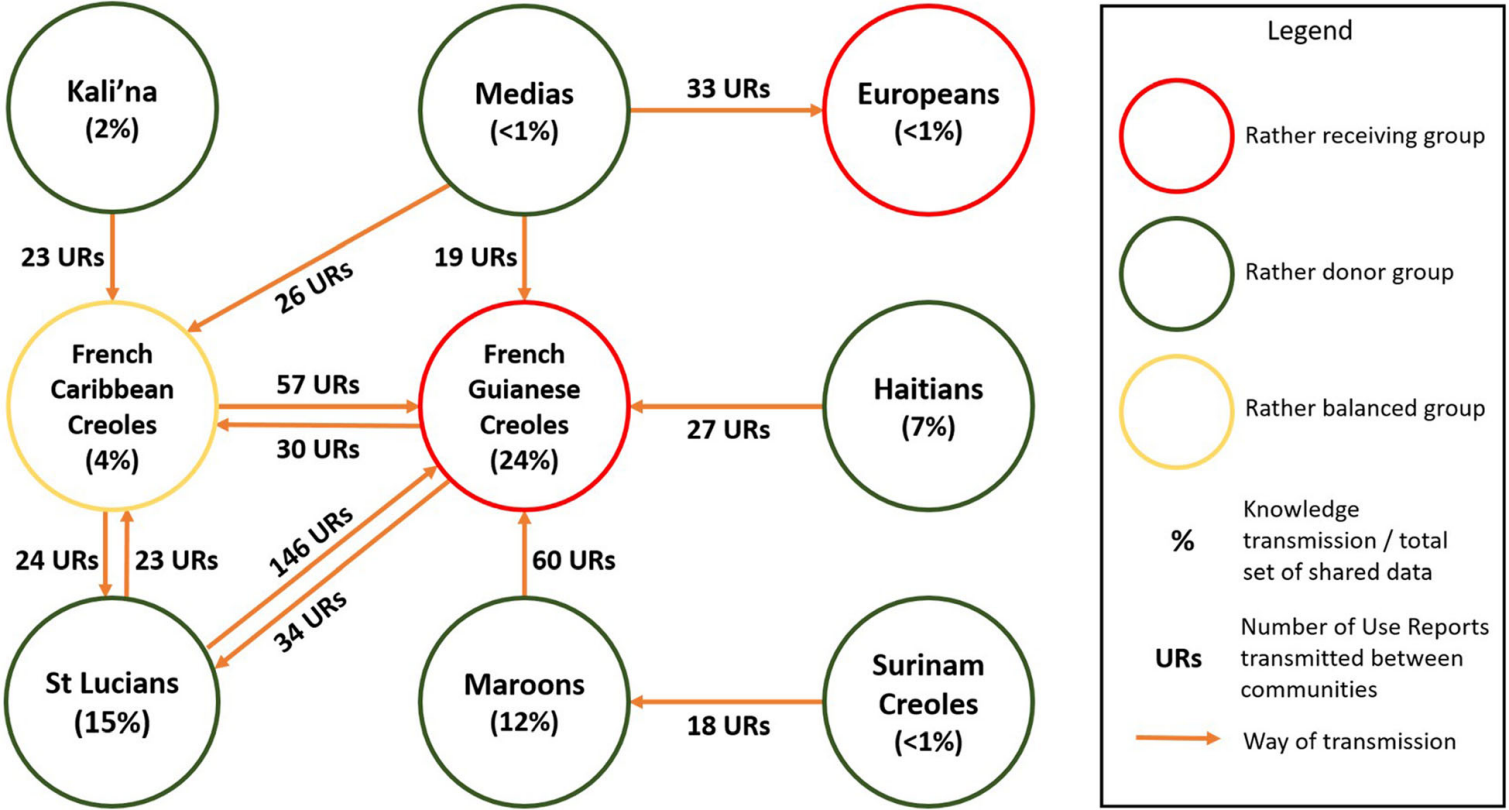
Fluxes of phyto-medicinal knowledge on the Guiana coast. Permeability/porosity and capacity for dissemination by the different communities interviewed

Then, in order to underline the diversity of intercultural exchanges, community profiles were drawn up for several groups, according to their level of permeability to exogenous knowledge and their ability to disseminate their own knowledge to other cultural groups. We first observe that this exchange web reveals inequalities both in terms of transmission and reception of knowledge (Fig. 4). Thus, the French Guianese Creoles and the Europeans seem to be relatively more assimilators of exotic knowledge while the Maroons, the St Lucians, and the Haitians transmit to other groups more than they receive from other communities.

**Fig. 5:**
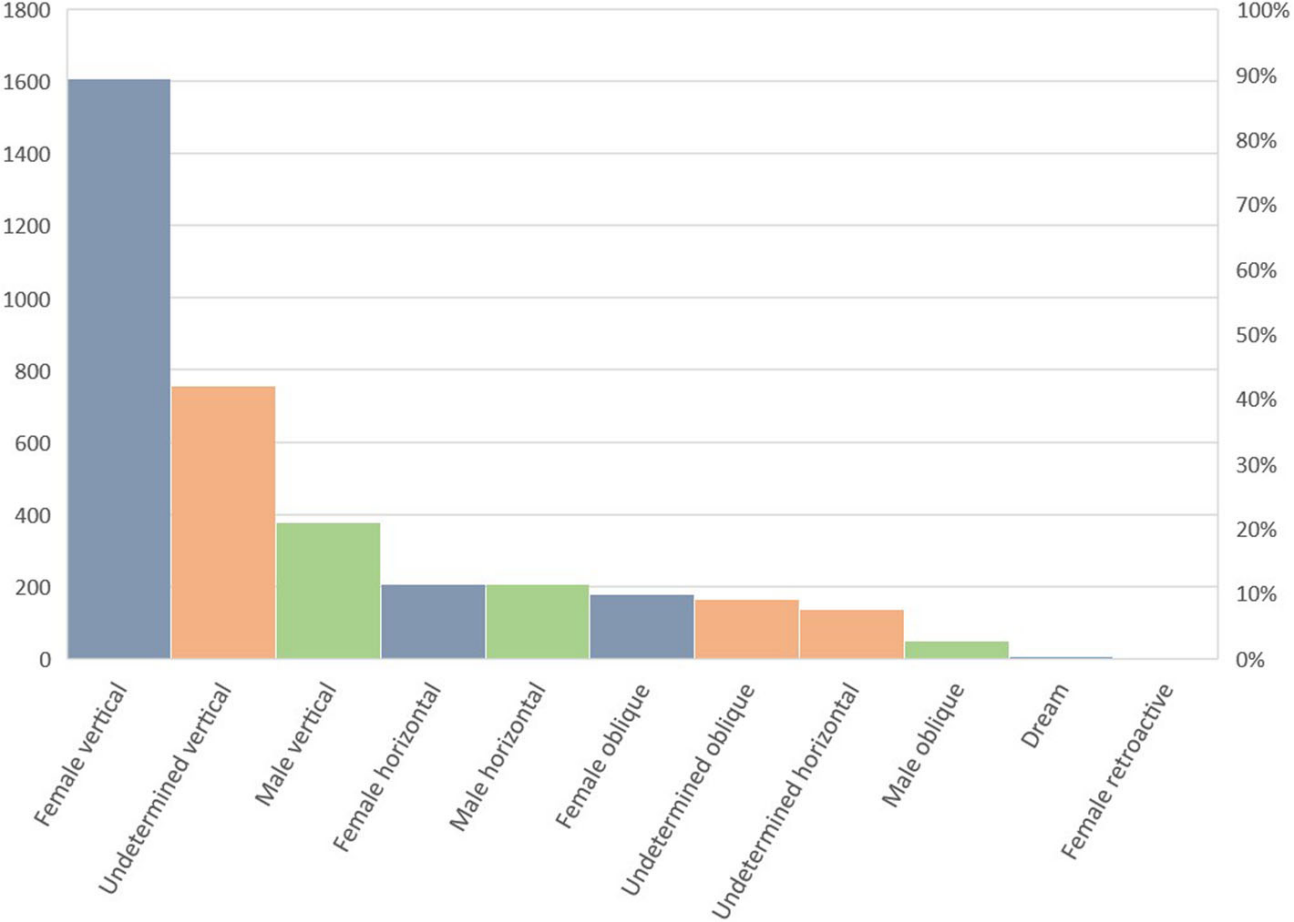
Proportion of the different types of transmission of herbal medicine knowledge on the French Guiana coast (URs and %)

More finely, when crossing the cultural affiliation of the respondents and the cultural affiliation of the people who transmitted to them the uses mentioned, it appears that the information is transmitted in more than 70% of the cases within the same cultural group, underlying that intercultural exchanges are a minority. However, this does not mean that intercultural exchanges are negligible since 14.8% of the uses of the plants mentioned were still transmitted horizontally (546/3683). Over a long period of time, it certainly influences the global patterns of knowledge. We also observe that the transmission of knowledge related to medicinal plants along the French Guiana coast remains essentially intra-community and intergenerational, corresponding mainly to a vertical type transmission (Fig. 5).

In fact, 74.5% of the uses listed (2743/3683) were transmitted vertically, either exclusively female (mother, grandmother, aunt: 43.7%, 1608/3683 URs), only male (father, grandfather, uncles: 10.3%, 379/3683 URs), or mixed (parents, grandparents: 14.7%, 546/3683 URs). Taking more widely into account every kind of transmission, women still hold a central place in the transmission of knowledge around medicinal plants in French Guiana since knowledge originating directly from women accounts for 54.1% of all transmissions (1993/3683), compared with 17.2% for those transmitted by men (632/3683 URs) and 28.6% from a mixed or undetermined gendered source (1053/3683 URs). In addition, if women transmit more than men, they also seem to be greater holders of knowledge since they total an average of 19.8 citations of plants (2355 URs for 119 women interviewed) against 15.4 for men (1328 URs for 86 men encountered).

## Discussion: distant influences as factors of a gradual interculturalization

### People interviewed

Our sampling takes into account the different socio-demographic components of the French Guianese society, in terms of age, gender, sectors of activity, and ethnicity. However, the important cultural diversity of French Guiana leads to a large number of smaller sub-groups whose number of people interviewed is sometimes insufficient to further analyze, with significant results, medicinal uses instead of species.

Moreover, the number of French Guianese Créoles interviewed may seem relatively disproportionate compared to the other groups present. Despite the fact that this group is still one of the most numerous minority (as counting for less than 50% of the overall population), it certainly reflects the importance of the creolization process which still seems to operate in French Guiana [42].

### Botany

A large number of the medicinal plants cited are cultivated, often as food or aromatic plants. To become popular, in addition to their intrinsic or supposed therapeutic properties, medicinal plants often need to be abundant and easily accessible [43–46]. Among the main botanical families cited in our study, several are also known to contain weeds (Asteraceae, Lamiaceae, Poaceae, Verbenaceae…), confirming this trend.

### Do plants travel with people, or people with plants?

As true as humans travel, plants do the same. Many medicinal plants have become, over time, “globalized plants” [47], marketed and consumed worldwide, even far from their areas of origin [48, 49]. As our study also shows, today’s pharmacopeias are therefore very globalized, containing many pantropical species. According to the “versatility theory” [50, 51], these are often species whose wide distribution seems linked to their primary use as ornamental plants [50, 52] or food [50, 52–55]. In addition, migrants who often already have a familiar relationship with these species in their country of origin continue to use them preferentially when they find them during their transnational movements, as has already been highlighted in several ethnobiological studies [3, 5, 9, 11]. But traveling plants may sometimes become invasive species, despite their medicinal properties [45, 46]. Indeed, according to the availability theory [46, 51, 56, 57], many medicinal plants are selected as such above all because they are abundant. Thus, this study also shows the weight of some invasive exotic species in French Guiana. If from an ecological point of view the arrival in a new environment of invasive species is unanimously recognized as negative for local biodiversity [58, 59], from a cultural point of view, on the other hand, more and more authors suggest taking into account the perception that local populations have of these species, particularly when they benefit of these species [58, 60–67]. The example of *Melaleuca quinquenervia* in French Guiana illustrates well this latter idea and shows how socially constructed the status of an “invasive” species can be, and remains extremely relative from a cultural point of view. Native to eastern Australia and presumably introduced in French Guiana during the middle of the twentieth century for the production of paper pulp [68], the species largely colonized the herbaceous savannas of the coast of French Guiana and tends today to extend in the east of Suriname. Conservationists perceive it as a “serious ecological problem” and recommend “to eradicate it absolutely” [69]. However, the Ndjuka populations of the Maroni basin have widely adopted it as a first-class medicinal species, thanks to its appreciated menthol scent (they call it *fekisi uwii*, literally “Vicks© leaves”) and its abundant dissemination which is perceived as “a blessing.”

### Cross-culturalism

As the intense cross-culturalism observed in the uses of the medicinal flora seems to show, intercultural exchanges are present and might eventually lead to a relative homogenization of phytotherapy practices. Very often, this pattern is reinforced by a “visualization process” effect [2] that could be assimilated to an “oblique” type transmission [43]. Overall, this result shows how questionable it seems for a community to appropriate the use of a particular plant, these being in most cases transversal to several cultural groups.

### Divergences and specificities

The observed fluxes of medicinal plant-related knowledge are also different in nature according to the groups. In accordance with the theory of “centrality” developed by Hopkins [70], the groups that are the most integrated into exchange networks are also those that are most inclined to intercultural exchange through interaction with other cultural communities. Milliken and Albert [71] also argue in this sense that the level of phytotherapeutic knowledge depends on the degree and nature of contact between neighboring socio-cultural groups and, in Guyana, van Andel et al. [72] show that the Arawak, having “a longer history of contact with the urban areas,” cite more exotic species than the Karib populations from the interior. In French Guiana, our observations therefore seem to confirm this trend: recent migrants (Haitians, Surinamese Creoles, Brazilians), less integrated in these interethnic contemporary exchange networks, cite generally a lower number of use reports (Table 1) and especially cite fewer local species. For example, the Amazonian *Bignoniaceae* species *Mansoa alliacea*, generally widely used in medico-magic remedies [39], is not used by any of the migrant communities while it is widely used among Amerindian, Maroon, and French Guianese Creole groups. One can however estimate, as postulated by Abreu et al .[21], that the children of migrants will in turn show a more hybrid knowledge, made of both knowledge acquired locally and knowledge specific to their group of origin and transmitted by their parents. This is undoubtedly what explains the high number of use reports of the St Lucian community (Table 1), which seems to have gathered over several generations very diversified uses thanks to multiple learning favored by a prolonged contact with the indigenous populations subsequent to the first gold rushes [73]. In addition, we can legitimately assume that this “gradual interculturalization” is also multilateral: migrant communities integrate over time by assimilating local customs but also by contributing to the pharmacopeias evolution through the introduction of new species and practices.

### Access strategies and transmission

The level of knowledge around medicinal plants seems favored by migratory contributions, and this even in the city, as already observed by Tareau et al. [16, 17] or by van Andel et al. [74]. Indeed, if the artificialization of rural spaces and the sedentarization of urban populations can appear as a major obstacle to the perpetuation of phytotherapy practices and knowledge [75], we note however that in average, levels of knowledge appear in French Guiana roughly similar among urban and non-urban residents. This result might be explained by two different ways. On the one hand, it is certainly linked to the fact that a large part of the urban dwellers being of recent rural origin (immigration, rural exodus), the link with nature seems stronger and is for the moment probably maintained in the city in some segments of the population, in which the phytotherapy practices still constitutes an inexpensive alternative to biomedicine and a strong identity marker [16, 17]. On the other hand, even in densely populated areas, contact with nature is still possible in the cities of French Guiana: people go picking on the Cayenne forested hills, in the “Malgaches’ forest” in St. Laurent, or in urban wastelands [17]. We were also able to show that many plants came from outside French Guiana along the migration process, which is consistent with the “relocalization” sub-process of ethnobotanical hybridizations described by Ladio and Albuquerque [22]. The influence of the Caribbean and bordering countries, from where a large part of the French Guianese population originates, is clearly highlighted here, showing the constructive role that migration plays on the evolution of the composition of the pharmacopeias. As observed elsewhere [5, 7, 76], migrant communities continue to use cultural keystone species, which are esteemed species (and products) that are firmly rooted in their original therapeutic traditions, relocating plants and practices in their country of settlement. In accordance with the previous observations of Tareau et al. [28], this phenomenon is particularly intense in “tinctorial” border areas which thus act as important zones of influence with regard to local populations.

This study once again highlights the predominant place of women in the transmission of ethnobotanical knowledge, in accordance with the literature [16, 77–83]. Our results are in line with other ethnobiological works which have shown that vertical and intra-cultural transmission constitutes the main mode of transmission of biological knowledge in many societies [43, 84–86]. This mode of transmission can also be considered more ethnocentric [87], favoring a certain compartmentalization of practices and a largely identical reproduction of parental practices by the following generations.

## Conclusion

Finally, what emerges from this study is above all that the multicultural context, characterized by significant migratory flows, generates a complex, polymorphous, and always dynamic medicinal flora. The pharmacopeias are continually enriched with new species and uses coming from diverse horizons, and each cultural group’s medicinal floras seem to overlap while sharing, in the same time, a number of common features. Indeed, behind a shared facade, strong cultural specificities nevertheless stand out through the existence of culturally indicative species. Nevertheless, this study is only based on the citations of species counted for each of the cultural groups, which hides another diversity of distinct medicinal uses.

The transmission of knowledge of herbal medicine, mainly feminine and intra-cultural, initially promotes a compartmentalization of knowledge and uses, which end up spreading over time, but in a differentiated way depending on the nature and the intensity of exchanges between communities. The relative centrality of some groups in intercultural exchanges therefore seems to be an important explanatory factor in the conservation or progressive erasing of culturally marked practices.

However, the context of interculturalism undeniably fosters continuous changes in herbal medicine practices. New plants and uses appear (or disappear) thanks to the movements and contacts of populations, perpetually redrawing the contours of dynamic pharmacopeias, built upon multiple and permanent influences. It would be interesting to go even further in this type of study by trying to understand how these changes operate at the scale of several generations, operating either a gradual leveling of intercultural knowledge or, on the contrary, maintaining a compartmentalization over time.

## Data Availability

Plants collected are deposited in the Cayenne IRD herbarium (CAY), under the voucher numbers MAT 01 to MAT 510. Interview data and consent forms are stored at the Laboratoire Ecologie, Evolutions, Interactions des Systèmes Amazoniens (LEEISA), Centre de Recherche de Montabo, Cayenne, French Guiana. The data contained in this paper is presented with more details in Tareau’s PhD dissertation [29].
